# Myeloid-specific deletion of chitinase-3-like 1 protein ameliorates murine diet-induced steatohepatitis progression

**DOI:** 10.1007/s00109-023-02325-4

**Published:** 2023-05-11

**Authors:** Andrea D. Kim, Lin Kui, Benedikt Kaufmann, Sung Eun Kim, Aleksandra Leszczynska, Ariel E. Feldstein

**Affiliations:** 1 Department of Pediatrics, University of California San Diego, 3020 Children’s Way, MC 5030, La Jolla, San Diego, CA 92103-8450 USA; 2 Department of Surgery, TUM School of Medicine, Klinikum rechts der Isar, Technical University of Munich, Munich, Germany; 3 Department of Internal Medicine, Hallym University College of Medicine, Chuncheon, Republic of Korea; 4 Institute for Liver and Digestive Diseases, Hallym University, Chuncheon, Republic of Korea

**Keywords:** Chitinase-like proteins, Non-alcoholic fatty liver disease, Hepatic stellate cells, Infiltrating macrophages

## Abstract

**Abstract:**

Chitinase-3-like 1 protein (CHI3L1) is a secreted glycoprotein, strongly correlated with fibrosis severity in chronic liver diseases including non-alcoholic steatohepatitis (NASH). However, the mechanisms by which CHI3L1 contributes to fibrogenesis remain undefined. Here, we showed that infiltrating monocyte-derived liver macrophages represent the main source of CHI3L1 in murine NASH. We developed a floxed CHI3L1 knock-out (KO) mouse to further study the cell-specific role of CHI3L1 ablation. Wildtype (WT) and myeloid cell-specific CHI3L1 KO mice (Cre^Lyz^) were challenged with a highly inflammatory and fibrotic dietary model of NASH by administering choline-deficient high-fat diet for 10 weeks. Macrophage accumulation and inflammatory cell recruitment were significantly ameliorated in the Cre^Lyz^ group compared to WT (F4/80 IHC *p* < 0.0001, CD11b IHC *p* < 0.0001). Additionally, hepatic stellate cell (HSC) activation and fibrosis were strongly decreased in this group (α-SMA IHC *p* < 0.0001, picrosirius red staining *p* < 0.0001). In vitro studies were performed stimulating bone marrow derived macrophages, THP-1 (human monocytes) and LX2 (human HSCs) cells with recombinant CHI3L1 to dissect its relationship with fibrosis development. Results showed an important role of CHI3L1 regulating fibrosis-promoting factors by macrophages (*TGFB1 p* < 0.05, *CTGF p* < 0.01) while directly activating HSCs (*ACTA2 p* < 0.01, *COL1A1 p* < 0.01), involving IL13Rα2 as the potential mediator. Our findings uncovered a novel role of CHI3L1 derived from liver macrophages in NASH progression and identifies this protein as a potential anti-fibrotic therapeutic target.

**Key messages:**

- We showed that CHI3L1 expression is increased in murine CDAA-HFAT diet NASH model, and that infiltrating macrophages are a key source of CHI3L1 production.
- Myeloid cell-specific CreLyz CHI3L1 knock-out in mice fed with CDAA-HFAT diet improved the NASH phenotype, with significantly reduced accumulation of pro-inflammatory macrophages and neutrophils compared with WT group.
- DEG and qPCR analysis of genes in CreLyz CHI3L1 knock-out mouse liver showed the mechanistic role of CHI3L1 in cellular chemotaxis.
- HSC is directly activated by CHI3L1 via receptor IL13Rα2, leading to upregulation of collagen deposition and pro-fibrotic gene, TIMP-1 and TIMP-2 release in whole liver.
- Direct stimulation of macrophages with CHI3L1 leads to upregulated expression of HSC-activation factors, suggesting its role in modulating macrophage-HSC crosstalk.

**Supplementary Information:**

The online version contains supplementary material available at 10.1007/s00109-023-02325-4.

## Introduction

Non-alcoholic fatty liver disease (NAFLD) is currently considered a major global health problem [1]. While becoming the most common chronic liver disease in the Western countries, the incidence of its progressive form, non-alcoholic steatohepatitis (NASH), has also increased, eventually leading to advanced liver disease and development of liver cirrhosis and hepatocellular carcinoma [2]. The main histopathological features that define NASH include hepatocellular damage, inflammation, and varying degrees of fibrosis through mechanisms that are intricately interconnected [3].

Chitinase-3-like protein 1 (CHI3L1) is a secreted glycoprotein, homologous to the breast regression protein-39 (BRP39) in mice and YKL-40 glycoprotein in humans. Although CHI3L1 belongs to the 18-glycosyl hydrolase family consisting of chitinases and chitinase-like proteins (CLP), it lacks the chitin-degrading activity due to a mutation in the active site [4, 5]. CHI3L1 is synthetized by a wide variety of cells including macrophages, neutrophils, and vascular smooth muscle cells, and shown to be an active contributor in the progression of chronic inflammatory disorders [4, 5]. Various inflammatory mediators such as IL-1 [4], IL-13 [6], IL-6, and TNF-α [4] act as CHI3L1 regulators. Recent studies have also shown the important role of CHI3L1 in cellular proliferation [7, 8], macrophage survival [9], inflammatory cell recruitment and differentiation [10], tissue remodeling, angiogenesis, and cancer metastasis [4, 5].

In humans, circulating levels of YKL-40 have been shown to be increased in various inflammatory-fibrotic disorders [7–10]. In the setting of chronic liver diseases, YKL-40 is currently being proposed as a promising diagnostic biomarker for liver fibrosis. Several reports demonstrated a close correlation between serum levels of CHI3L1 and severity of hepatic fibrosis in NAFLD [11–13], viral hepatitis [14–16], and ALD [17]. Currently, YKL-40 has been incorporated as a novel serum biomarker for the assessment of fibrosis staging in both hepatic and extrahepatic diseases [18–22].

Although a direct correlation between CHI3L1 serum concentrations and liver fibrosis stages has been well established in NAFLD [12, 13], the mechanisms by which CHI3L1 contributes to the progression of NASH and liver fibrosis remain incompletely understood. In this study, we determined the source and contribution of CHI3L1 on NASH development through cellular and molecular mechanisms by using various in vivo and in vitro approaches including a human-relevant male murine model of progressive fibrotic NASH choline-deficient, L-amino acid-defined, high-fat diet (CDAA-HFAT) and genetically modified mice with cell-specific deletion of the CHI3L1 gene.

## Results

### Hepatic expression and function of CHI3L1 (YKL-40) are increased in the CDAA-HFAT-induced murine fibrotic NASH model

**Fig. 1:**
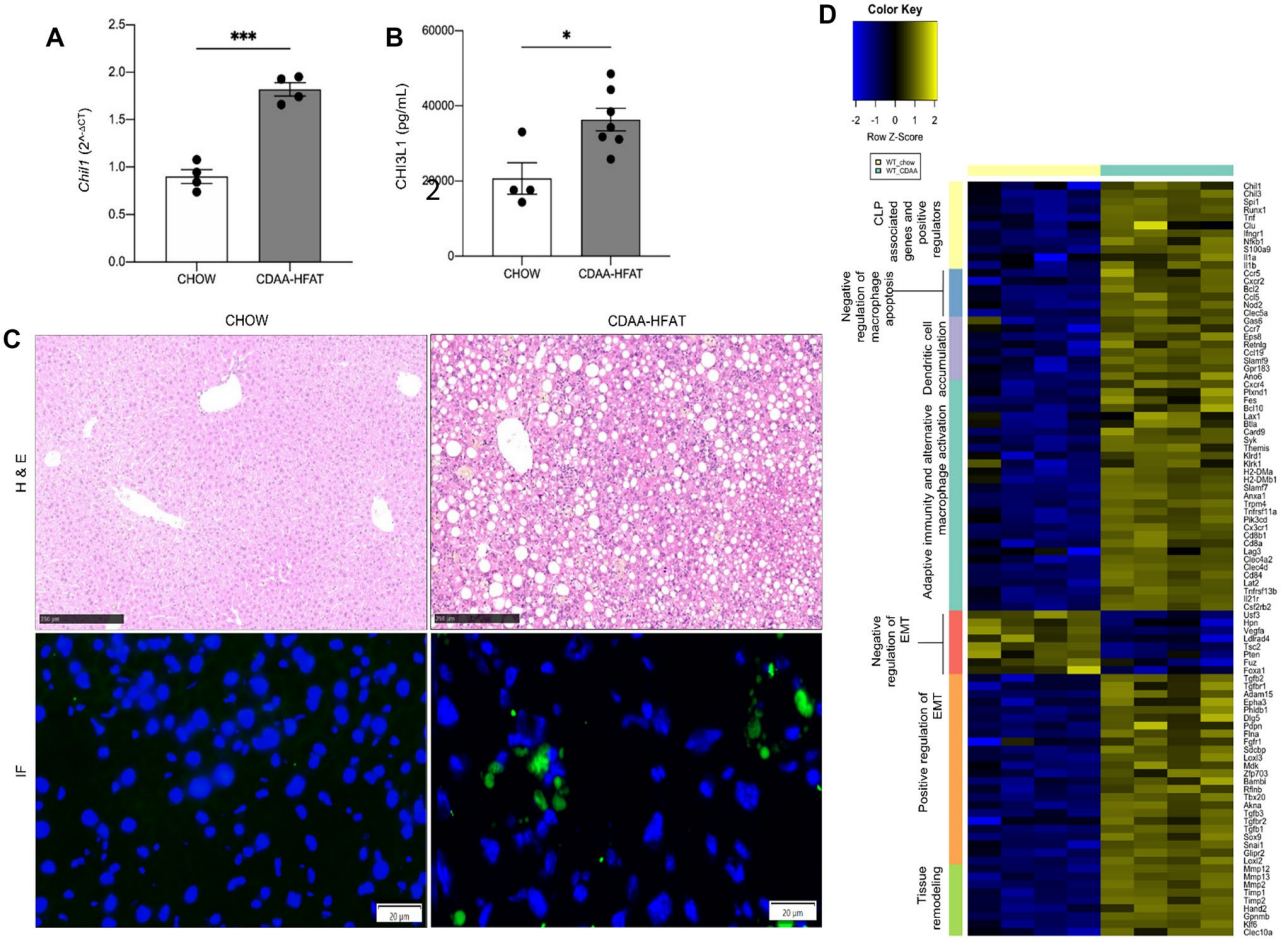
Hepatic expression of CHI3L1 levels are increased in murine CDAA-HFAT diet-induced NASH model. **A** RT-qPCR for the assessment of CHI3L1 coding gene *Chil1* expression in liver tissue was performed. **B** Release of systemic CHI3L1 in NASH and control mice was measured by serum ELISA. **C** H&E staining on CDAA-HFAT-receiving mice livers and immunofluorescence detection (IF) of CHI3L1 (scale bar: 20 μm) on respective frozen tissues were performed. **D** Heatmap representation of results from RNA bulk sequencing followed by differential expression of genes (DEG) analysis of CDAA diet-induced NASH mice vs. chow diet controls is shown

We initially assessed the expression levels of CHI3L1 in a murine NASH model induced by the administration of a 10-week regimen of CDAA-HFAT diet to 8-week-old male C57BL/6 WT mice. A significant increase in the CHI3L1 coding gene *Chil1* expression (*p* < 0.001) was present in livers of CDAA-HFAT-fed mice (Fig. 1A), as well as augmented levels of CHI3L1 systemic release measured by serum ELISA (*p* < 0.05) (Fig. 1B). A directly correlating degree of NASH-induced inflammation was observed on H&E stainings, along with severe macrovesicular steatosis and cellular infiltration (Fig. 1C). Immunofluorescence detection of CHI3L1 was performed in frozen liver sections showing a characteristic non-hepatocytic perivascular pattern of expression compared to significantly lower detection signal in control mouse livers (Fig. 1C).

Additionally, we performed bulk RNA sequencing and differentially expressed genes (DEG) analysis in livers from control and CDAA-HFAT-fed mice. In our murine NASH model, we found a significant enrichment of genes related to the promotion of chitinase-like proteins (*Spi1*, *Tnf, Ifngr1, Il1b*) as well as CHI3L1 downstream signaling pathways including induction of Th2 inflammation, tissue remodeling, epithelial-to-mesenchymal transition (EMT) and alternative activation of macrophages (Fig. 1D).

These data demonstrated an evident upregulation of CHI3L1 in murine fibrotic-NASH. Consistent with reports in patients with NASH, an increased in circulating CHI3L1 protein in serum could be observed. RNA sequencing and DEG analysis showed a significant enrichment of pathways involving the function of CHI3L1, suggesting a high level of activity of this protein in our CDAA-HFAT-induced NASH model.

### CHI3L1 is expressed in infiltrating Ly6C^hi^ macrophages and myeloid cell-specific deletion of CHI3L1 (Cre^Lyz^) results in decreased gene expression and release of CHI3L1

**Fig. 2:**
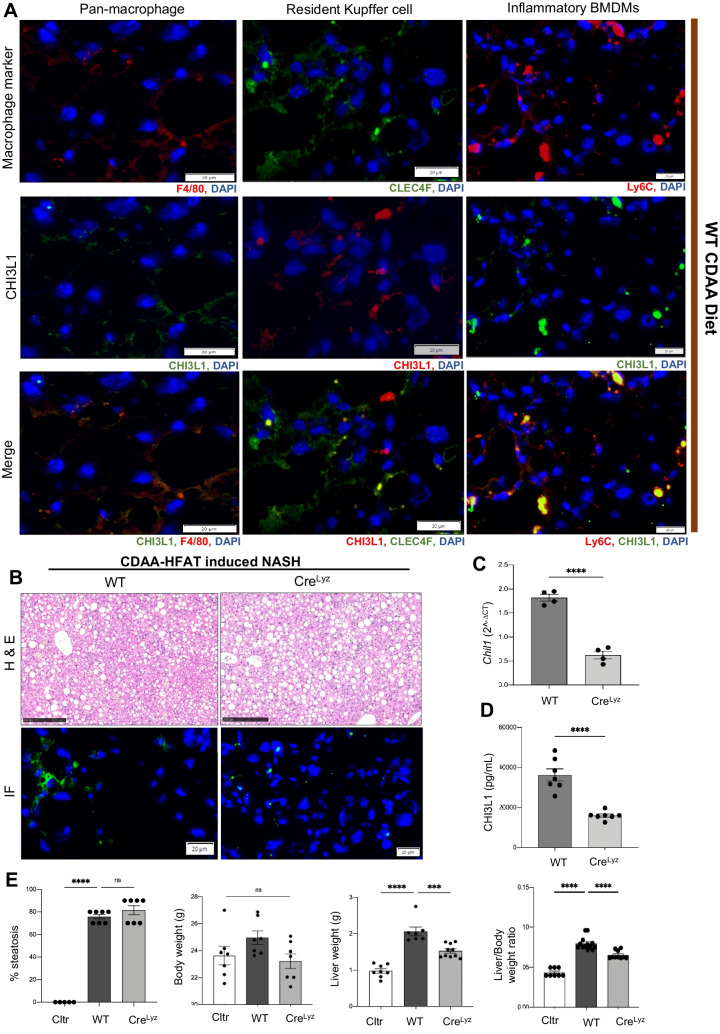
Infiltrating BMDMs represent the main source of CHI3L1 in CDAA-HFAT-induced NASH. **A** Immunofluorescence detection of macrophage subset markers and CHI3L1 were used to co-localize the specific source of CHI3L1 in murine diet-induced NASH liver of WT mice. Pan-macrophage marker F4/80, hepatic resident Kupffer cell marker CLEC4F and infiltrating pro-inflammatory marker Ly6C were merged with CHI3L1 signaling (scale bar: 20 µm). **B** H&E staining (scale bar: 250 μm) and IF detection of CHI3L1 (scale bar: 20 μm) were performed in WT and Cre^Lyz^ mice challenged with 10 weeks of CDAA-HFAT diet. Hepatic gene expression (**C**) and protein release into serum (**D**) of CHI3L1 were measured in the previous groups via RT-qPCR and ELISA respectively. **E** Degree of steatosis, body weights, liver weights and liver/body ratios were compared between control, WT and Cre^Lyz^ groups on CDAA-HFAT diet

Macrophages were identified as a main source of CHI3L1 production in numerous reports [7, 23, 24]. In our model, this was consistent with the main immunofluorescent detection of CHI3L1 in perivascular areas but none in parenchymal areas occupied by hepatocytes. When co-localization of CHI3L1 and pan-macrophage marker F4/80 was performed, an evident overlap between the two markers supported macrophages as the main CHI3L1 source in our CDAA-HFAT-induced NASH model. To identify the specific macrophage subset responsible for CHI3L1 production, we verified if detection of CHI3L1 co-localized with resident (CLEC4F^+^) or infiltrating (Ly6C^+^) macrophage markers (Fig. 2A). When merging these signals, a significant overlap between CHI3L1 and Ly6C^+^ macrophages was observed, while a lack of co-localization could be seen in resident CLEC4F^+^ macrophages, suggesting that infiltrating activated Ly6C^+^ macrophages are the main source of CHI3L1 during NASH development, rather than the resident Kupffer cells.

Therefore, we next compared the NASH phenotype when CHI3L1 is selectively deleted in myeloid cells. Genetically modified C57BL/6 mice containing the vector with the exon 5 of the CHI3L1 gene as the conditional knock out region were generated and bred with pairs expressing Cre recombinase under the control of the myeloid cell-specific marker, lysozyme (Cre^Lyz^) expression. In order to confirm the correct selection of the conditional knock-out region and effectivity of the cell-specific deletion, we compared the basal expression of the *Chil1* gene in macrophage colony stimulating factor (MCSF)-stimulated bone marrow cells from WT and Cre^Lyz^ mice. Expression of CHI3L1 by Cre^Lyz^ was markedly decreased in comparison to WT mice (*p* < 0.01) (Supp. Fig. 1A, B).

Cre^Lyz^ mice were placed on CDAA-HFAT diet for 10 weeks. While no evident changes were observed in H&E staining in regards of degree of steatosis, immunofluorescence in liver frozen tissue showed an important decrease of CHI3L1 detection signal in Cre^Lyz^ mice compared to WT controls (Fig. 2B). *Chil1* gene expression was measured via RT-qPCR and serum protein levels of CHI3L1 were measured via ELISA. *Chil1* expression in the liver (*p* < 0.0001) and protein release into the serum (*p* < 0.0001) were diminished in this group (Fig. 2C, D). On immunofluorescence, CDAA-HFAT fed CreLyz mice livers showed decreased detection of Ly6C + macrophages, and almost absent detection of CHI3L1 in frozen sections (Supp. Fig. 1C). These results were also compared to an additional control group expressing a Cre recombinase under the control of albumin expression (Cre^Alb^), therefore resulting in hepatocyte-specific CHI3L1 K.O (Supp. Fig. 2A). CDAA-HFAT-induced NASH Cre^Alb^ mice showed no significant differences in hepatic expression of CHI3L1 gene (*p* = 0.125) and protein expression levels in serum (*p* = 0.065) compared to WT mice (Supp. Fig. 2B).

These results demonstrated that myeloid cells constitute the main source of CHI3L1 production in our model of CDAA-HFAT diet-induced NASH. While differences in the degree of steatosis and weight were not apparent (*p* = 0.335 and 0.114, respectively), a significant decrease of liver weight (*p* < 0.001) and liver/body weight (*p* < 0.0001) ratio was evidenced in the Cre^Lyz^ group (Fig. 2E). Differences in NASH-related inflammation and fibrosis progression were further compared between WT and Cre^Lyz^ mice.

### Myeloid-specific deletion of CHI3L1 results in significantly decreased NASH-related inflammatory cell accumulation in the liver

**Fig. 3:**
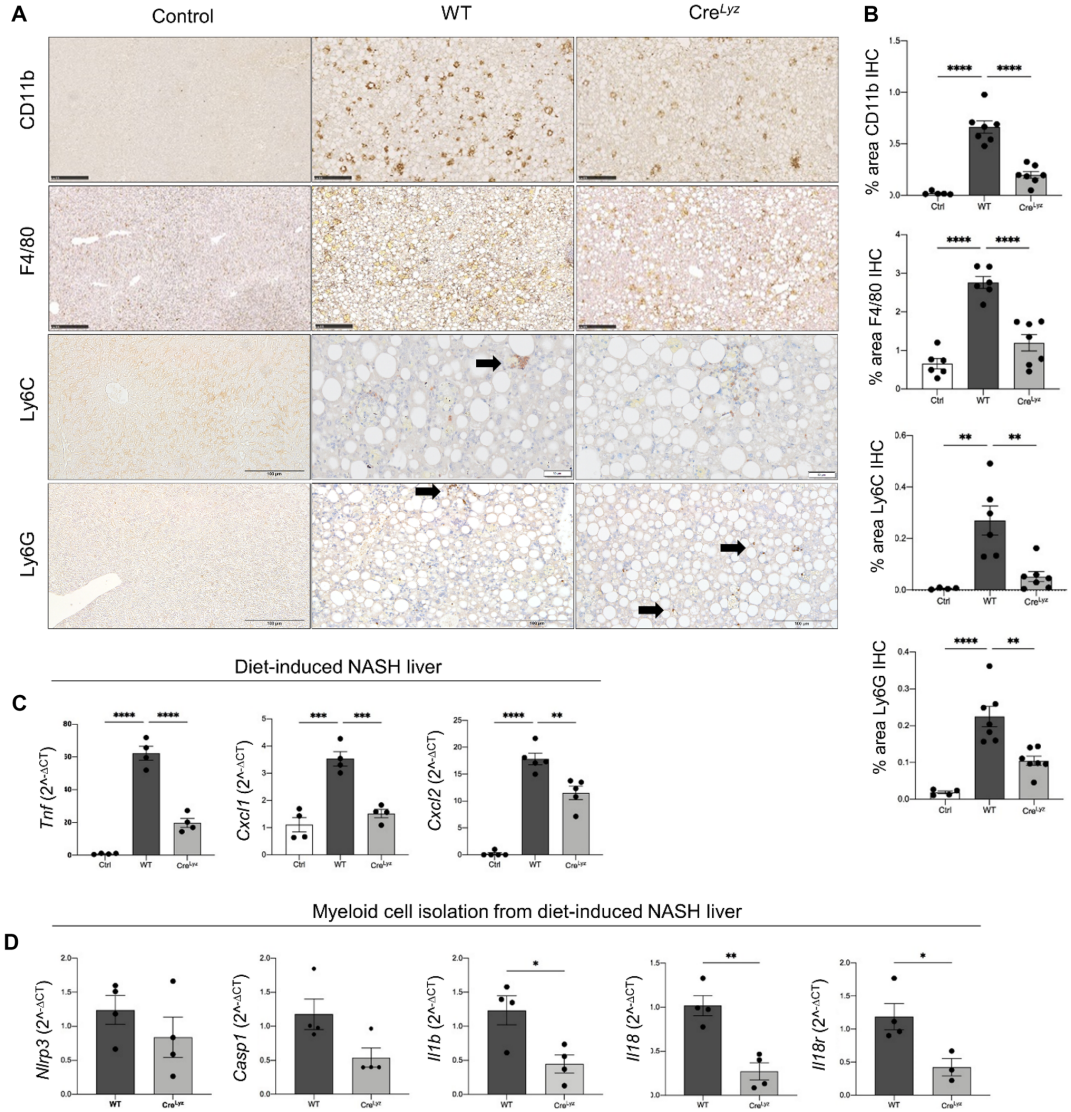
Myeloid cell-specific deletion of CHI3L1 abrogates NASH-related inflammatory cell infiltration and activity of pro-inflammatory macrophages and neutrophils. **A**, **B** Infiltrating immune cells were detected by CD11b immunohistochemistry (scale bar: 250 μm). Both total and inflammatory macrophages were measured by F4/80 (scale bar: 250 μm) and Ly6C (control scale bar: 100 μm, WT and Cre^Lyz^: 50 μm) IHC respectively, and presence of neutrophils was measured via Ly6G detection (control scale bar: 100 μm, WT and Cre^Lyz^: 50 μm). Differences were quantified and compared between the control and NASH-induced WT and Cre^Lyz^ groups. **C** mRNA expression of pro-inflammatory marker *Tnf* and main neutrophil chemoattractants *Cxcl1* and *Cxcl2* were measured by RT-qPCR in livers from the mentioned groups. **D** Myeloid cells were isolated via enzymatic liver perfusion and density gradient centrifugation from CDAA diet-receiving WT and Cre^Lyz^ mice. Markers of the NLRP3 inflammasome activation pathway were measured by RT-qPCR of these cell fractions

CD11b-positive infiltrating immune cells were significantly decreased in IHC of paraffin-embedded liver sections in Cre^Lyz^ group (*p* < 0.0001) compared to WT liver section. Total macrophages and pro-inflammatory activated macrophages were detected by performing F4/80 (*p* < 0.0001) and Ly6C (*p* < 0.01) IHC, respectively. Mature neutrophils were also measured via the recognition of the specific marker Ly6G in paraffin-embedded liver sections (*p* < 0.01), with perivascular cluster-like infiltration in WT mice (Fig. 3A). The Cre^Lyz^ group showed a significant reduction of all these populations when compared to WT CDAA-HF-fed mice (Fig. 3B). These changes were not evidenced when comparing the WT group to the hepatocyte-specific CHI3L1 KO group (*p* = 0.763 CD11b IHC, 0.294 F4/80 IHC, 0.349 Ly6C IHC, and 0.229 Ly6G IHC) (Supp. Fig. 2C). Consistently, mRNA expression of pro-inflammatory cytokine *Tnf* (*p* < 0.0001) and main neutrophil chemoattractants *Cxcl1* (*p* < 0.001) and *Cxcl2* (*p* < 0.01) were measured via RT-qPCR in liver biopsies showing a significant decrease in the myeloid cell-specific CHI3L1 KO group (Fig. 3C).

Further focused assessment of the myeloid compartment changes during NASH development was done by performing an enzymatic hepatic perfusion followed by isolation of myeloid cells in WT and Cre^Lyz^ mice. mRNA levels of the NLRP3 inflammasome pathway components were analyzed as it functions as an essential component of inflammation and fibrosis development in NASH [25]. Although *Nlrp3* (*p* = 0.314) was not significantly different between WT and Cre^Lyz^ groups, *Casp1* ((*p* = 0.053) showed a considerably decreased expression trend, along with significant downregulation of downstream mediators *Il1b* (*p* < 0.05) and *Il18* (*p* < 0.01). Gene expression of *l18r* (*p* < 0.05) was also observed to be significantly diminished in the Cre^Lyz^ myeloid cells (Fig. 3D).

In summary, Cre^Lyz^ mice showed significantly diminished infiltration of inflammatory cells, resulting in a decreased accumulation of pro-inflammatory activated Ly6C^+^ macrophages and neutrophils. These data suggest that myeloid cell-mediated release of CHI3L1 contributes to the development of inflammatory cell accumulation and activity in NASH by being involved in the production of pro-inflammatory mediators, neutrophil chemoattractants, and inflammasome-mediated activation of IL-1β and IL-18.

### Cre^Lyz^ mice showed significantly ameliorated activation of HSC and decreased fibrosis compared to WT in CDAA-HFAT diet-induced NASH

**Fig. 4:**
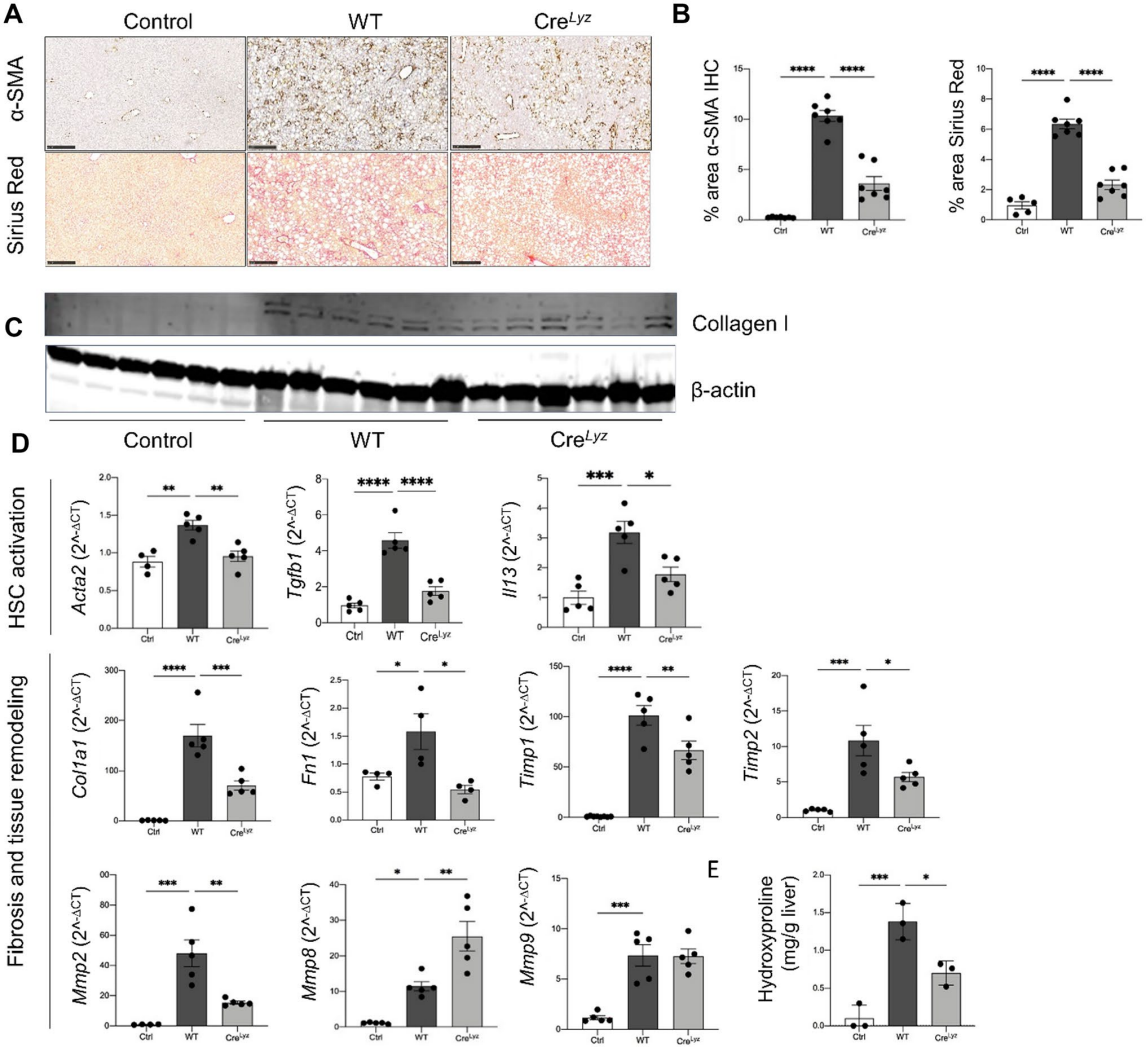
Myeloid cell-specific deletion of CHI3L1 ameliorates HSC activation and liver fibrosis in CDAA-HFAT-induced murine NASH. **A**, **B** Hepatic stellate cell activation marker α-SMA and collagen were detected via IHC and Sirius Red staining in liver paraffin-embedded sections (scale bar: 250 µm). **C** Western blotting of liver lysates for detection of collagen type 1. Entire blotting shown in Supp. Fig. 4. **D** mRNA expression of HSC activation markers (*Acta2*, *Tgfb1 and Il13*), fibrosis markers (*Col1a1, Fn1, Timp1, Timp2, Mmp2*) as well as collagen degrading matrix metalloproteinases (*Mmp8* and *Mmp9*) were measured in liver tissue. **E** Hydroxyproline assay was performed and collagen synthesis levels were compared among the control, WT and Cre^Lyz^ groups

Hepatic stellate cell activation and the formation of extracellular matrix proteins leading to fibrosis are key indicators of disease progression in NASH. Alpha smooth muscle actin (α-SMA), a marker of activated HSC was detected by IHC and RT-qPCR in liver tissue, showing a significantly decreased signal in the Cre^Lyz^ group (*p* < 0.0001 and < 0.01, respectively) when compared to WT (Fig. 4A, B, and D). Consistently, gene expression of mediators of HSC activation, TGF- and IL-13 [26], were both significantly down regulated in the Cre^Lyz^ group (*p* < 0.0001 and 0.05, respectively).

Collagen deposition was evaluated via picro-sirius red (*p* < 0.0001) stainings, collagen type 1 western blotting (full-length western blot in Supp. Fig. 3A), mRNA expression (*p* < 0.001) and quantification of hydroxyproline in liver tissue (*p* < 0.05) (Fig. 4A, B, C, D, and E). Significant amelioration of collagen synthesis was shown in the Cre^Lyz^ group. Furthermore, mRNA expression of additional fibrosis-related proteins such as fibronectin-1 (*Fn1*, *p* < 0.05), tissue inhibitor of metalloproteinases (*Timp1 p* < 0.01, *Timp2 p* < 0.05), and matrix metalloproteinase-2 (*Mmp2, p* < 0.01), measured by RT-qPCR, showed a significant decrease in the Cre^Lyz^ group compared to WT. An opposite upregulation or non-significant changes were observed for the expression of collagen-degrading *Mmp8* (*p* < 0.01) and *Mmp9* (*p* = 0.995) genes, respectively, both known to play an important role in the inflammation resolution phase of NASH (Fig. 4D).

When these markers were similarly evaluated in the CDAA-HFAT fed Cre^Alb^ group (α-SMA IHC *p* = 0.8, picrosirius red *p* = 0.99), no significant difference could be appreciated in the modulation of NASH-related HSC activation and fibrosis deposition when compared to the WT group (Supp. Fig. 2C).

### In vitro studies showed both direct and indirect effects of CHI3L1 on HSC activation

**Fig. 5:**
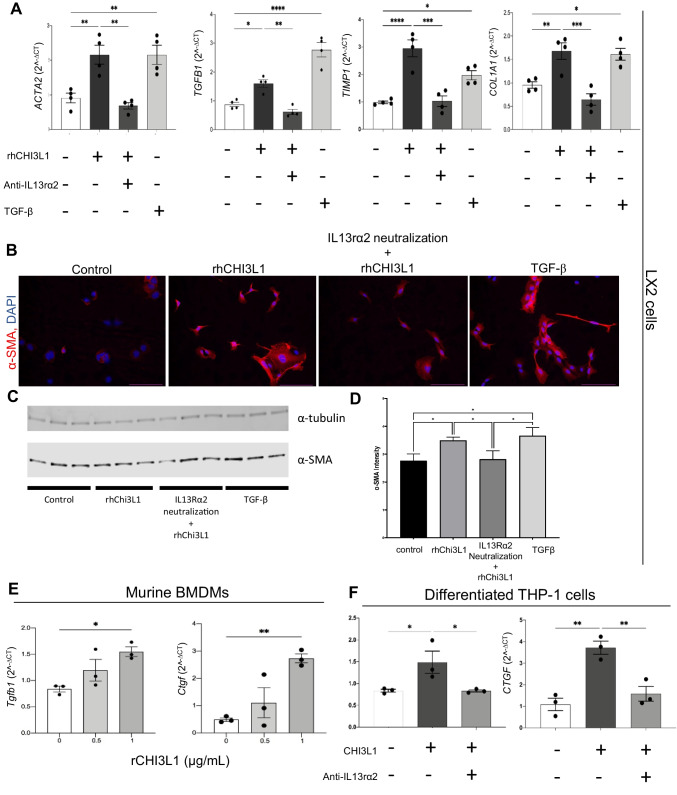
Stimulation with recombinant CHI3L1 demonstrates direct and indirect IL13rα2-mediated mechanisms on HSC activation. **A** LX2 (human HSCs) cells were stimulated with 100 ng/mL of human recombinant CHI3L1 (rhCHI3L1). A separate group was pre-treated with neutralizing anti-IL13rα2 polyclonal antibodies prior to rhCHI3L1 stimulation. Recombinant TGF-β was used as a positive control. HSC activation markers were measured by RT-qPCR. **B** HSC-activation marker, α-SMA detection was evidenced via immunocytochemistry (20X), Westernblot (**C**) and quantification of α-SMA intensity (**D**). **E** Differentiated bone marrow macrophages were stimulated with 0.5 and 1 μg/mL of murine recombinant mouse CHI3L1 (rCHI3L1) and mRNA expression of HSC activators *Tgfb1* and *Ctgf* were measured by RT-qPCR. **F** Differentiated THP-1 (human macrophages) cells were stimulated with rhCHI3L1 with and without pre-treatment with neutralizing anti-IL13rα2 polyclonal antibodies and expression of *TGFB1* and *CTGF* were measured by RT-qPCR. Values were expressed as the mean of duplicates of 3 independent experiments

Based on the differences observed in HSC activation and fibrosis between WT and the Cre^Lyz^ group, we proceeded to evaluate the direct effect of CHI3L1 on HSC activation. We cultured human HSCs (LX2 cell line), followed by stimulation with 100 ng/mL of recombinant human CHI3L1 (rhCHI3L1) or equal volumes of PBS for 24 h while stimulation with TGF-β was used as a positive control. The measurement of HSC activation-related marker genes expression via RT-qPCR showed significantly increased expression levels of *ACTA2* (*p* < 0.01), *TGFB1* (*p* < 0.05), *TIMP1* (*p* < 0.0001), and *COL1A1* (*p* < 0.01) (Fig. 5A). Consistently, activation of HSCs was determined via immunocytochemistry and western blotting (full length WB in Supp. Fig. 3B, C), whereby a higher intensity of α-SMA was detected in HSCs stimulated with rhCHI3L1 (Fig. 5B, C, and D). However, when neutralization of IL-13 receptor α2 (IL13rα2), a well-described receptor for the mediation of tissue remodeling effects by CHI3L1, preceded the stimulation, a significant decrease was observed in the expression of HSC activation markers *ACTA2* (*p* < 0.01), *TGFB1* (*p* < 0.01), *TIMP1* (*p* < 0 0.001), and *COL1A1* (*p* < 0.001), close to the levels of non-stimulated cells. These results suggest that CHI3L1 regulates canonical HSC activation via IL13rα2-mediated effects.

The clear association between CHI3L1 and HSC activation and subsequent fibrosis prompted us to evaluate if additional indirect mechanisms contribute to the activation of HSCs. As the main source of HSC-stimulating mediators consists of infiltrating immune cells such as macrophages which activate the inflammatory milieu in NASH [27], we evaluated if CHI3L1 would upregulate the release of pro-fibrotic factors from these cells.

Bone marrow myeloid cells from WT mice were plated and differentiated into macrophages. Differentiated macrophages were subsequently stimulated with 0.5 and 1 µg/mL of mouse recombinant CHI3L1 or equal volumes of PBS for 24 h. Factors related to HSC activation and fibrosis were measured via RT-qPCR, and showed a gradual dose-dependent increase of *Tgfb1* and *Ctgf,* main HSC-activating factors, achieving statistical significance at the concentration of 1 µg/mL of rCHI3L1 (*p* < 0.05 and < 0.01, respectively) (Fig. 5E). These results were also observed with the stimulation of human macrophage line THP-1 cells with 100 ng/mL of rhCHI3L1 (*TGFB1 p* < 0.05 and *CTGF p* < 0.01) (Fig. 5F). The increase of expression of these factors was similarly abolished when preceded by the neutralization of IL13rα2 (*TGFB1 p* = 0.993, *CTGF p* = 0.547). These results suggest CHI3L1 holds an additional indirect mechanism on HSC activation by binding to macrophages via IL13rα2 and promoting the release of profibrogenic factors. The basis behind these findings still needs to be elucidated. One possible explanation could be the effect of CHI3L1 skewing the macrophages phenotype into M2 (upregulating the expression of CCL22), as it can be observed in Supp. Fig. 4.

**Fig. 6:**
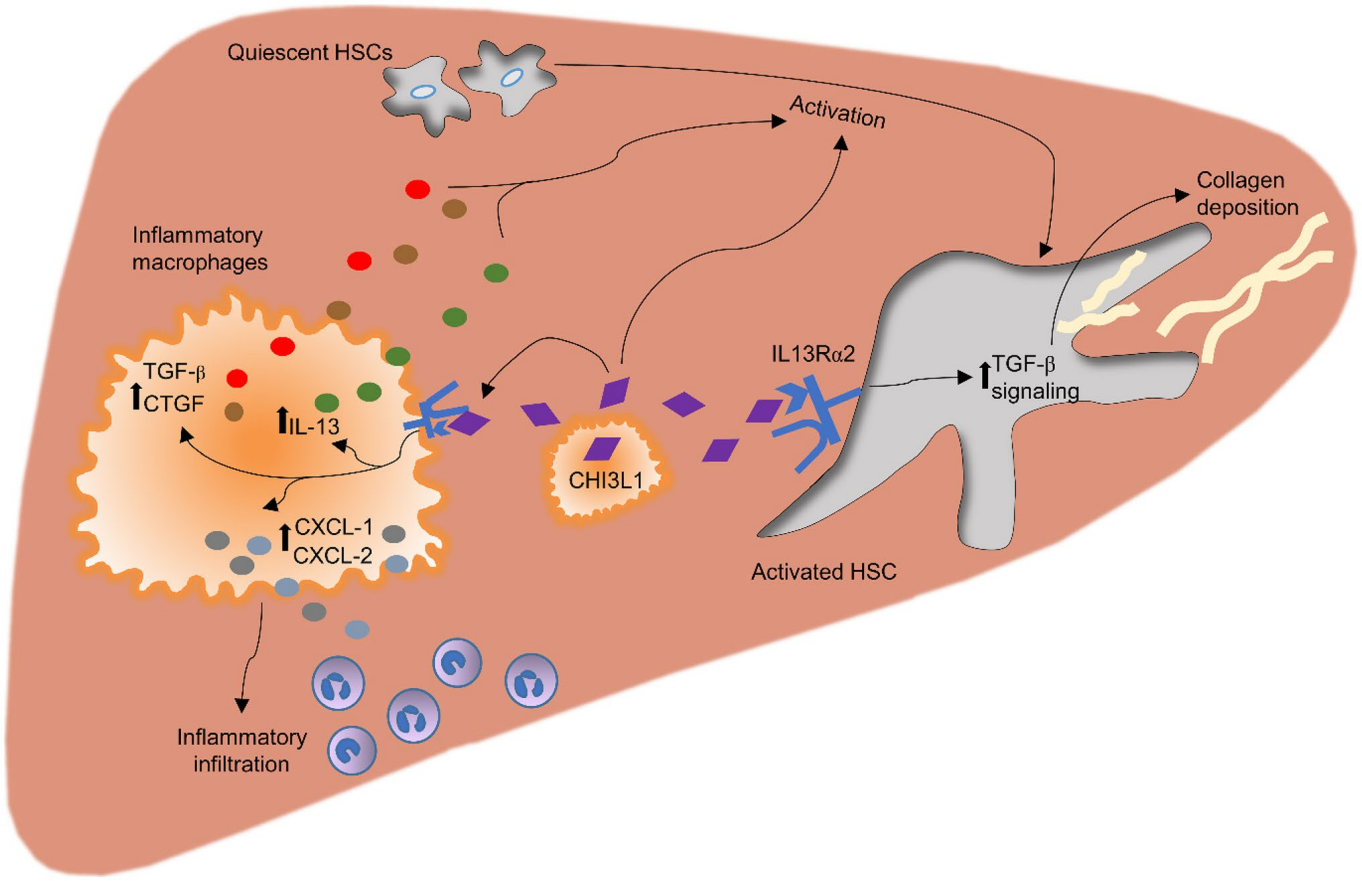
Proposed mechanism of CHI3L1 involvement in the progression of CDAA-HFAT diet induced NASH fibrosis and inflammation. Infiltrating pro-inflammatory Ly6C^hi^ macrophages demonstrated to be a crucial source of CHI3L1 in diet-induced NASH model. Release of CHI3L1 from these cells have a direct effect in the macrophage-HSC crosstalk. HSCs are directly stimulated by CHI3L1 by binding to its receptor, IL-13 receptor alpha 2 (IL13Rα2). The activation of the later induce the activation of TGF-β mediated pathways which enhance quiescent HSC differentiation into myofibroblasts with augmented production of extracellular matrix and collagen deposition. On the other hand, CHI3L1 holds additional ways of indirectly stimulating this transformation. CHI3L1 stimulates macrophages in an autocrine manner and release of HSC activators such as TGF-β and CTGF ensues. An important increase in the production and release of IL-13 further activates HSCs. CHI3L1 additionally serves as an activation signal for the release of chemotactic mediators such as CXCL-1 and CXCL-2 that results in the crucial recruitment and accumulation of pro-inflammatory cells, resulting in positive feedback loop

In conclusion, CHI3L1 is not only an important direct HSC activator, but could hold additional effects by stimulating the surrounding immune cells to release HSC-activating factors and pro-fibrotic mediators (Fig. 6).

## Discussion

Liver fibrosis is an intrinsic response to chronic persistent liver injury that results in not only a wound-healing process to mitigate damage, but also scar formation leading to the development of cirrhosis. This results in disruption of the liver architecture and vascular distortion, associated with severe liver-related complications including portal hypertension, liver failure, and hepatocellular carcinoma. Nonalcoholic steatohepatitis (NASH), the progressive form of non-alcoholic fatty liver disease (NAFLD), has become increasingly common worldwide over the last two decades. Patients with advanced liver fibrosis are at significantly increased risk of liver-related morbidity and mortality for which no effective therapeutic intervention has yet been approved. Therefore, identifying novel mechanistic drivers of liver fibrosis in the context of NASH is imperative for the development of novel treatments.

Circulating levels of CHI3L1 (YKL-40) have been proposed as a robust non-invasive biomarker of liver fibrosis that allows for staging and diagnosis of fibrosis without the need of a liver biopsy [12, 13]. Studies have demonstrated that serum concentrations of YKL-40 are able to differentiate early from late stages of liver fibrosis in NAFLD patients [12, 13]. Because of its value as a diagnostic biomarker for liver fibrosis, elucidation of the biological functions of CHI3L1 and potential mechanistic contribution to the development and progression of NASH-related inflammation and fibrosis are warranted.

We first set out to investigate the changes in expression levels and cellular sources of CHI3L1 in the context of NASH by using a murine model that recapitulates the features of advanced fibrotic human NASH. The results showed an increase of CHI3L1 levels in murine NASH, identifying infiltrating activated macrophages as the main source of production. Therefore, we next explored the role of cell-specific CHI3L1 deletion by developing a novel floxed CHI3L1 mouse. A myeloid cell-specific (Cre^Lyz^) CHI3L1 knock-out mice was generated and compared to a Cre recombinase-lacking (WT) group. We additionally generated a hepatocyte-specific (Cre^Alb^) CHI3L1 KO group to further assess the potential role of hepatocytes as a source of CHI3L1 during NASH. In our study, clear abrogation of immune cell accumulation including pro-inflammatory macrophages and neutrophils representing key cellular mediators of NASH progression was observed in the Cre^Lyz^ CHI3L1 KO group on the CDAA-HFAT diet compared to WT group. In a previous study in a murine chronic renal fibrosis model due to ischemia–reperfusion injury, CHI3L1 was shown to be chronically released in small amounts from damaged epithelial cells and involved in macrophage persistence [7]. Higashiyama et al. found that CHI3L1 enhanced macrophage survival by suppressing Fas expression and activating Akt signaling in an autocrine manner, resulting in hampered macrophage apoptosis [23], suggesting that the lesser accumulation of macrophages in our Cre^Lyz^ could be due to increased cell death in this population. Additionally, in the setting of colorectal cancer, CHI3L1 showed to be crucial for the maximal enhancement of inflammatory chemotaxis mediated by the release of IL-8 and MCP-1 from cancer cells [24]. Consistently, our results also supported the contribution of CHI3L1 in cellular chemotaxis by showing a significant downregulation of *Cxcl1* and *Cxcl2* in our Cre^Lyz^ mice livers. Pulmonary inflammation models have linked CHI3L1 released from macrophages to the inflammatory skew into Th2 inflammation by releasing IL-4, IL5, IL-13, and promoting the accumulation of dendritic cells and macrophage with alternative activation [8, 28]. These findings were consistent with the DEG analysis results, where CDAA-HFAT-induced NASH mice showed significant upregulation of CHI3L1 regulators along with adaptive immunity, M2 macrophage polarization, and dendritic cell accumulation-related genes.

Additionally, we found a significant difference in the HSC activation levels and collagen deposition between WT and Cre^Lyz^ groups on the CDAA-HFAT diet. CHI3L1 has been proposed as an important stimulator of human connective tissue cells and described to be involved as a pro-fibrotic biomarker in numerous hepatic and extrahepatic chronic disorders [7, 12, 13, 23, 29]. Direct activation of HSCs has been evidenced by us and others as one of CHI3L1 functions [30]. Activation of IL13Rα2, well known for the mediation of CHI3L1 downstream effects, has been described to activate TGF-β-pathways [6, 26], leading to a rise in collagen deposition and pro-fibrotic TIMP-1 and TIMP-2 release, as seen in our results. These changes could not be observed with a previous neutralization of IL13rα2 receptors on the human HSC cell line (LX2) suggesting a direct HSC activating mechanism mediated by this receptor.

Moreover, previous reports have shown CHI3L1 to be required for the maximal expression of profibrotic growth factors PDGF-β and TGF-β1 from macrophages in a renal ischemia–reperfusion injury [7]. In idiopathic pulmonary fibrosis models, CHI3L1 demonstrated to be crucial for the accumulation of CD206^+^ macrophages that subsequently induce fibroblastic proliferation and survival [9]. Consistent with these findings, when differentiated bone marrow derived macrophages were stimulated in vitro with recombinant CHI3L1, HSC activating *Tgfb1*, and *Ctgf* genes were significantly upregulated in a dose-dependent manner. A similar increase in the expression of HSC-activating factors could also be evidenced with the stimulation of macrophages differentiated from the human monocytic cell line THP-1. These findings suggest that CHI3L1 not only holds direct HSC-activating properties but also plays an important indirect role by modulating the macrophage-HSC crosstalk.

CHI3L1 production has been described in numerous cell types in a pathology- and time-dependent manner [7]. The generation of cell-specific CHI3L1 knockout mice allowed us to mechanistically study in vivo the relevant cellular sources of CHI3L1 in the context of fibrotic NASH. This new tool will also aim the study of CHI3L1 in various other pathologies where a key role for this protein has been described. Additionally, our findings have identified important novel mechanisms involving CHI3L1 in inflammatory cell chemotaxis and accumulation as well as direct and indirect roles on HSC activation. The findings shed new lights into the role of CHI3L1 during NASH progression and identified CHI3L1 as a novel potential therapeutic target for NASH treatment.

## Methods

### Cell-specific CHI3L1 knock-out mice

To investigate the cell-specific role of CHI3L1 in diet-induced NASH, a CHI3L1 conditional knockout mouse model was generated. First, a linearized vector was engineered. Exon 5 on mouse chromosome 1 was targeted as conditional knock out region, as deletion of this region results in a loss of function. Mouse genomic fragments containing homology arms and the conditional knockout region were generated and amplified from bacterial artificial chromosome clone and sequentially assembled into a targeting vector together with recombination sites and selection markers (Supplement Fig. 2A).

In the targeting vector, the Neo cassette was flanked by SDA (self-deletion anchor) sites. DTA was used for negative selection. The linearized vector was transfected into C57BL/6 embryonic stem cells that afterwards were injected into C57BL/6 embryos and re-implanted into CD-1 pseudo-pregnant females. Founder animals were identified by their coat color and germline transmission was confirmed by breeding with C57BL/6 females and subsequent genotyping of the offspring (performed by Cyagen, Santa Clara, CA). Finally, the knock-out allele is obtained after certain Cre-mediated recombination. In this study, conditional CHI3L1 knockout mice were bred to mice expressing Cre recombinase under control of lysozyme (Cre^Lyz^) (myeloid lineage-specific knockout of CHI3L1, obtained from The Jackson Laboratory, Jax #004,781, B6.129P2-Lyz2tm1(cre)Ifo/J) and albumin (Cre^Alb^) (hepatocyte-specific knockout of CHI3L1, obtained from The Jackson Laboratory, Jax #003,574, B6.Cg-Speer6-ps1Tg(Alb-cre)21Mgn/J). Littermates lacking the Cre recombinase were used as control mice.

### Choline-deficient, L-amino acid-defined hight-fat diet-induced NASH

Eight-week-old male mice were fed choline-deficient, L-amino acid-defined high-fat diet (CDAA-HFAT, Research Diets, New Brunswick, NJ, USA) for 10 weeks to induce severe steatohepatitis with high levels of inflammation and fibrosis [31]. WT controls were fed with chow diet for the same period of time. Mice were euthanized by being placed in a chamber with a controlled carbon dioxide filling rate (50% of the chamber volume per minute) followed by cervical dislocation. All efforts were made to minimize pain and distress during animal husbandry and experimental assessments. This work was performed under adherence to the ARRIVE guidelines.

### Serum sample and liver preparation

Blood was extracted by performing cardiac puncture and coagulated at room temperature for 30 min. Serum was obtained after centrifugation at 300 rpm for 7 min and immediately stored at −80°.

Whole liver was extracted. Liver weight and liver/body weight ratio values were measured. Tissue was distributed in the following manner: two representative sections were immediately fixed in 10% formalin for 24 h and embedded in paraffin. A representative portion was embedded in O.C.T for future frozen section; samples of 50 μg were placed in 0.5 mL of RNAlater Solution (Lifetechnologies, Carlsbad, CA) for future RNA isolation in liquid nitrogen and stored at −80°.

### Histopathology and immunohistochemistry

Liver samples fixed in 10% formaldehyde and 70% ethanol for 24 h each were embedded in paraffin for microtome sections. Deparaffinized and rehydrated 5 µm sections were stained with hematoxycilin (Sigma-Aldrich, St. Louis, MO) and eosin (Richard Allan, Kalamazoo, MI) for steatosis evaluation. To detect fibrosis, sections were incubated for 1 h at RT with an aqueous solution of saturated picric acid (Sigma-Aldrich) mixed with 0.1% Fast Green fetal calf serum (FCS) and 0.1% Direct Red Dye (Sigma-Aldrich). Five randomly selected fields (×10 magnification) were photographed and the percentage of Sirius Red-stained area was measured by ImageJ software with an adjusted unchanged threshold.

For immunohistochemical staining, 5 µm thickness sections were deparaffinized and rehydrated with xylene and decreasing ethanol concentrations and distilled water. Sections were incubated for 5 min in 3% hydrogen peroxide for internal peroxidase blockade. For α-SMA and F4/80 detection, antigen retrieval was performed by incubating the sections in TBS-T with 2% BSA + 1% Triton X-100 for 30 min. For the remaining, this step was performed by incubating the sections in preheated Citrate Buffer pH 6.0 (Dako Reagents) in a 95 °C bath for 20 min. Sections were incubated with rabbit anti-α-SMA (ab124964, 1:500, Abcam), anti-CD11b (ab128797, 1:350, Abcam), rat anti-Ly6G (Cat 14–5931-82, 1:200, Invitrogen), anti-Ly6C (ab15627, 1:200, Abcam), or anti-F4/80 (Cat 123,106, 1:100, BioLegend) primary antibodies diluted in Dako Antibody Diluent (Odense, Denmark). After overnight incubation with the primary antibodies for 16 h, sections were washed with TBS-T and incubated with ready-to-use HRP-linked anti-rat or rabbit secondary immunoglobulin G antibody (Immpress HRP reagents; Vector Labs, Burlingame, CA) for 1 h at RT. Color was developed with DAB solution (Vector Labs) and nuclei was counterstained with Mayer’s hematoxylin for 2 min, followed by dehydration with increasing ethanol concentrations. Stainings were quantified in 5 randomly selected fields (×10 magnification) imaged with a Nanozoomer 2.0HT slide Scanner microscope (Hamamatsu Photonics K.K., Hamamatsu, Japan). The total stained area was analyzed by selecting brown areas using an unchanged threshold value in the macro function of ImageJ (NIH, Bethesda, MD, USA). Results were represented as the average of the percentage of total area occupied by positive cells per field in each specimen.

### Immunofluorescence of frozen mouse liver sections

Frozen liver sections were washed and fixed with ice cold methanol for 10 min. Next, the cells were washed, permeabilized (0.1% Tween-20 in PBS, 30 min), blocked for 1 h with 1% BSA-PBS and incubated at 4 °C with mouse monoclonal rabbit anti-CHI3L1 (ab180569, 1:100, Abcam), mouse anti-F4/80 (Cat 123,106, 1:100, BioLegend) and anti-Ly6C (ab15627, 1:100, Abcam), and goat anti-CLEC4F (AF2784, 1:100, R&D Systems) antibodies diluted in Dako Antibody Diluent. After overnight incubation, cells were washed and treated with Alexa Fluor 488 goat anti-rabbit (1:1000; Invitrogen), Alexa Fluor 594 goat anti-mouse (1:1000), Alexa Fluor 488 donkey anti-goat (1:1000) or Alexa Fluor 594 donkey anti rabbit (1:1000) for 1 h at RT in the dark, followed by washing and 5 min of nuclei staining with DAPI diluted at 1:1000 in PBS.

### Real-time PCR

Liver tissue underwent RNA extraction with TRIzol Reagent (Sigma-Aldrich) and 50 ug of purified RNA was reverse-transcribed into cDNA by using the qScript cDNA Synthesis Kit (Quantabio). The following gene primer sequences (Table 1) were used. Target gene expression levels were calculated with normalization to the *Gapdh/GAPDH* gene expression levels followed by a comparative cycle threshold Ct method (2^−ΔΔCt^).

**Table 1:** Gene primers used for mRNA expression analysis

	**Gene primers**	**Identifier (Taqman)**
Mouse	Acta2	Mm00725412_s1
	Casp1	Mm00438023_m1
	Chil1	Mm00657889_mH
	Col1a1	Mm00801666_g1
	Ctgf	Mm01192933_g1
	Cxcl1	Mm04207460_m1
	Cxcl2	Mm00436450_m1
	Fn1	Mm01256744_m1
	Gapdh	Mm99999915_g1
	Il1b	Mm00434228_m1
	Il13	Mm00434204_m1
	Il18	Mm00434226_m1
	Il18r1	Mm00515178_m1
	Mmp2	Mm00439498_m1
	Mmp8	Mm00439509_m1
	Mmp9	Mm00442991_m1
	Nlrp3	Mm00840904_m1
	Tgfb1	Mm01178820_m1
	Timp1	Mm01341361_m1
	Timp2	Mm00441825_m1
	Tnf	Mm00443260_g1
Human	ACTA2	Hs00426835_g1
	COL1A1	Hs00164004_m1
	CTGF	Hs00170014-m1
	GAPDH	Hs99999905_m1
	TIMP1	Hs01092512_g1
	TGFB1	Hs00998133_m1
	CCL22	Hs01574247_m1
	CXCL10	Hs00171042_m1

### Generation of bulk RNA-seq data from WT mice on CDAA and CHOW diet

Total RNA was assessed for quality using an Agilent Tapestation 4200, and 800 nanograms of RNA from samples with an RNA Integrity Number (RIN) greater than 8.0 were used to generate RNA sequencing libraries using the Illumina^®^ Stranded mRNA Prep (Illumina, San Diego, CA). Samples were processed following manufacturer’s instructions. Resulting libraries were multiplexed and sequenced with 100 basepair (bp) paired end reads (PE100) to a depth of approximately 25 million reads per sample on an Illumina NovaSeq 6000. Samples were demultiplexed using bcl2fastq Conversion Software (Illumina, San Diego, CA).

Quality control of the raw fastq files was performed using the software tool FastQC1 v0.11.8. Sequencing reads were trimmed with Trimmomatic2 v0.38 and aligned to the mouse genome (GRCm38.p63) using the STAR aligner4 v2.5.1a. Read quantification was performed with RSEM5 v1.3.0 and the Ensembl release 98 annotation6. The R BioConductor packages edgeR7 and limma8 were used to implement the limma-voom9 method for differential expression analysis. In brief, lowly expressed genes–those not having counts per million (cpm) e 1 in at least 4 of the samples–were filtered out and then trimmed mean of M-values (TMM)10 normalization was applied. The experimental design was modeled upon condition. representing genotype and diet (~0 + condition). The voom method was employed to model the mean–variance relationship in the log-cpm, after which lmFit was used to fit per-gene linear models and empirical Bayes moderation was applied with the eBayes function. Significance was defined by using an adjusted *p*-value cut-off of 0.05 after multiple testing correction11 using a moderated t-statistic in limma. Functional enrichment of the differentially expressed genes was performed using WebGestalt12 (including GSEA13), and gProfiler14.

### CHI3L1 Enzyme-linked immunosorbent assay (ELISA)

Mouse serum CHI3L1 was measured in 50 μL of diluted serum (1:20) with the mouse Quantikine CHI3L1 ELISA Kit, following the manufacturer’s instructions (Cat no. MC3L10, RnD Systems).

### Isolation of murine NASH liver myeloid cells

Mice were anesthetized by ketamine/xylazine injection. Livers were separately digested in situ by perfusion through the infrahepatic inferior vena cava with EGTA, pronase E (0.4 mg/ml), collagenase D (0.8 mg/ml) for 5 min each as previously described [32]. Each liver was placed individually in ice-cooled enzyme buffer solution until simultaneous in vitro digestion with pronase and collagenase D solution (containing 1% DNAse) on a stirring plate for 24 min at 40 °C. Sample was processed through a 70 μm cell strainer and hepatocytes were removed via centrifugation at 500 rpm for 3 min 2 times. Non-parenchymal cells were centrifuged at 2000 rpm for 8 min and resuspended with GBSS-B in addition to DNase I. A 3-layer cell gradient was separated using a Nycodenz gradient centrifugation. Myeloid cell fraction was collected between the upper and medium gradient, washed in PBS and prepared for RNA isolation and RT-qPCR.

### Bone marrow derived cells isolation and recombinant CHI3L1 stimulation

Bone marrow cells were collected by flushing femurs with DMEM 1% HEPES, 1% Penicillin–Streptomycin, 1% non-essential amino acids and 10% FBS. The sample was filtered through a 40 μm mesh and centrifuged at 1500 rpm for 10 min RT. Pellet was resuspended with media, plated at 2 × 10^6^/cm2 and differentiated into macrophages with macrophage colony simulating factor (MCSF) for 5 days at 37 °C, 5% CO2, and 85–98% humidity. Stimulation with mouse recombinant CHI3L1 (Cat 2649-CH, R&D Biosystems) was performed at 0, 0.5, and 1 μg/mL at day 5 for 24 h and cells were harvested and prepared for RNA isolation for RT-qPCR or protein isolation for western blotting.

### Cell culture

Human monocyte-like cells (THP-1) (tib-202, ATCC) were plated at 10^6^/cm^2^ cells in DMEM 1% Penicillin–Streptomycin, 2 mM L-glutamine, 25 mM HEPES, 10% FBS. Cells were differentiated into macrophages with 10 ng/mL of phorbol 12-myristate 13-acetate (P139-1MG, Millipore-Sigma) for 48 h and rested with media for 24 h. After differentiation, cells were stimulated with 100 ng/mL of recombinant human CHI3L1 (rhCHI3L1, 2599-ch, R&D Biosystems)) or equivalent amount of PBS for 24 h. A separate group was pre-treated with 2 μg/mL of polyclonal anti-IL13Rα2 neutralizing antibodies (Cat. PA5-46,976, Invitrogen) for 6 h prior to the stimulation with rhCHI3L1. Cells were harvested and prepared for RNA isolation for RT-qPCR.

Human hepatic stellate cells (LX2, kindly provided by Prof. Scott Friedman) were plated at 5 × 10^4^/cm2 cells in DMEM 1% Penicillin–Streptomycin, 10% FBS. At day 2, cells were stimulated with 100 ng/mL of recombinant human CHI3L1 or equivalent amount of PBS for 24 h. A separate group was pre-treated with 2 μg/mL of polyclonal anti-IL13Rα2 neutralizing antibodies for 6 h prior to the stimulation with rhCHI3L1. Stimulation with 2.5 ng/mL of recombinant TGF-β (Cat. 100–21, Peprotech) for 24 h was used as a positive control. Cells were harvested and prepared for RNA or protein isolation for RT-qPCR or western blotting, respectively.

For immunocytochemistry, 5 × 10^2^/cm^2^ of LX2 cells were plated overnight on a Nunc Lab-Tek II 4-well chamber slide (Thermo Fisher Scientific) to allow attachment and differentiation at 37 °C, 5% CO2, and 85–98% humidity. Cells were washed and fixed with ice cold methanol for 10 min. Cells were washed, permeabilized (0.2% Tween-20 in PBS, 30 min), blocked for 1 h with 1% BSA-PBS, and incubated at 4 °C with mouse monoclonal anti-α-SMA (ab124964, 1:500, Abcam) antibody diluted in PBS. After overnight incubation, the cells were washed and treated with Alexa Fluor 488 anti-rabbit (1:1000; Invitrogen) for 1 h at RT in the dark, followed by washing and 5 min of nuclei staining with DAPI diluted at 1:1000 in PBS. The chamber was then removed, and the cells were mounted on a cover glass with Dako Fluorescent Mounting Media.

### Immunoblot analysis

Protein fraction was purified from approximately 100 mg of liver biopsy using RIPA lysis buffer for 40 min on ice. Twenty-five microgram of total tissue protein were loaded in SDS with 3% mercaptoethanol, heated for 5 min at 95 °C, electrophoresed via an Any kDa precast Tris–Glycine (Bio-Rad) at 80 V for 30 min, followed by 120 V until the end of the run. Tris–glycine-SDS was used as running buffer. Proteins were transferred from the gel into a nitrocellulose membrane in a Trans Blot Turbo Transfer system (Bio-Rad) for 7 min at 25 V. An ethanol based solution was used as transfer buffer (Bio-Rad), followed by blockage of the membrane for 1 h with the Intercept Blocking Buffer (Li-Cor) at RT.

Membranes was sectioned to avoid cross-reaction and incubated with mouse monoclonal anti β-actin (1:5000, A5441-2ML, Sigma) and goat anti-collagen type 1 (1:50, 1310–01, Southern Biotech) primary antibodies at 4 °C overnight. Membrane was washed two times in TBS-tween and incubated with HRP-linked anti-mouse or goat IgG antibodies, respectively (1:15.000, Li-Cor) for 1 h at RT. Protein bands were scanned with the Odissey Infrared Imager (Li-Cor) and analyzed with the Image Studio software.

### Hepatic hydroxyproline quantification

One hundred milligram of liver was homogenized and hydrolyzed by being baked at 110 °C for 18 h with 6N HCl. Homogenates were filtered and aliquots were evaporated at 60° for 40 min. Crystals were resuspended with 50 μL of water and incubated for 20 min with 100 μL Chloramine-T solution (C9887, Sigma) at room temperature. One hundred microliter of Ehrich’s reagent (D2004, Sigma) was then added and samples were incubated at 65 °C for 20 min. Absorbance was measured at 558 nm. Standard seriated curve was made using stock trans-4-hydroxy-L-proline (Sigma, Cat#7279).

### Statistics

Statistical analyses were performed with Graph Pad Prism (version 7; Graph Pad Software Inc., La Jolla, CA, USA). The significance of 2-group comparisons was determined with 2-tailed Student’s *t* test. The significance of more than 2 groups was analyzed by 1-way ANOVA, corrected by Tukey’s post hoc test. *p* values less than 0.05 were considered significant. Error bars represent means ± SEM. Experiments were repeated at least 3 times, and assays were performed in duplicate.

### Supplementary Information

Below is the link to the electronic supplementary material.

Supplementary file1 (PPTX 3278 KB)

Supplementary file2 (XLSX 1437 KB)

## Data Availability

The dataset generated during and/or analyzed during the current study will be made available in the supplementary information as required.
