# Correction to: Myeloid‑specific deletion of chitinase‑3‑like 1 protein ameliorates murine diet‑induced steatohepatitis progression

**DOI:** 10.1007/s00109-023-02388-3

**Published:** 2023-11-12

**Authors:** Andrea D. Kim, Lin Kui, Benedikt Kaufmann, Sung Eun Kim, Aleksandra Leszczynska, Ariel E. Feldstein

**Affiliations:** 1 Department of Pediatrics, University of California San Diego, 3020 Children’s Way, MC 5030, La Jolla, San Diego, CA 92103‑8450 USA; 2 Department of Surgery, TUM School of Medicine, Klinikum rechts der Isar, Technical University of Munich, Munich, Germany; 3 Department of Internal Medicine, Hallym University College of Medicine, Chuncheon, Republic of Korea; 4 Institute for Liver and Digestive Diseases, Hallym University, Chuncheon, Republic of Korea

**Correction to: Journal of Molecular Medicine (2023) 101:813–828** **https://doi.org/10.1007/s00109-023-02325-4**

**Conflict of Interest** Ariel E. Feldstein is currently (since July 2022) an employee and stockholder of Novo Nordisk.

