# Supplementary material for: Myeloid-specific deletion of chitinase-3-like 1 protein ameliorates murine diet-induced steatohepatitis progression
Source: J Mol Med (Berl). 2023 May 11;101(7):813–28. doi: 10.1007/s00109-023-02325-4 (PMC10300183; doi:10.1007/s00109-023-02325-4)
Supplement: Supplementary file 1 — Supplementary file1 (PPTX 3278 KB) [file 109_2023_2325_MOESM1_ESM.pptx]

## Slide 1
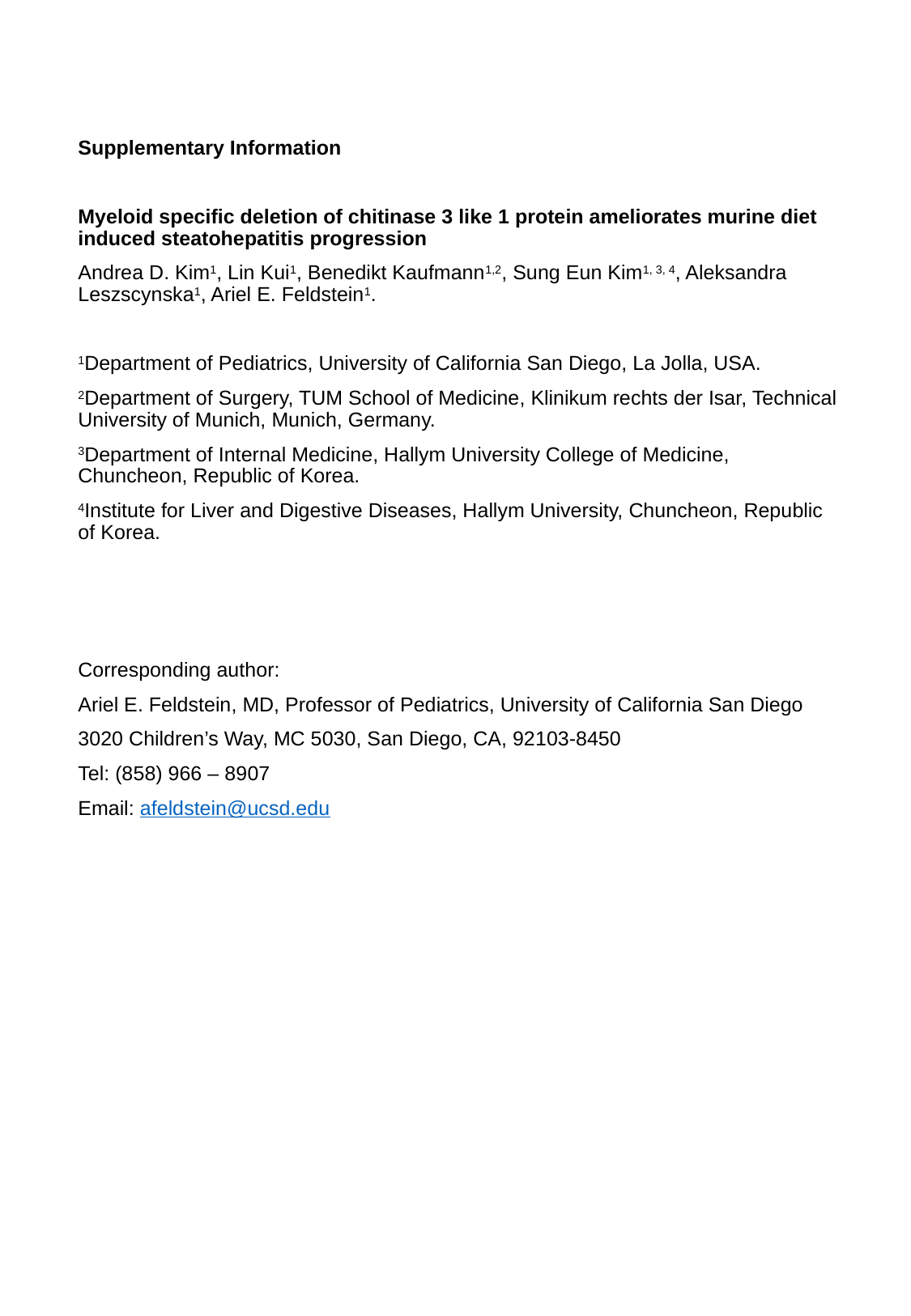

Supplementary Information
Myeloid specific deletion of chitinase 3 like 1 protein ameliorates murine diet induced steatohepatitis progression
Andrea D. Kim1, Lin Kui1, Benedikt Kaufmann1,2, Sung Eun Kim1, 3, 4, Aleksandra Leszscynska1, Ariel E. Feldstein1.
1Department of Pediatrics, University of California San Diego, La Jolla, USA.
2Department of Surgery, TUM School of Medicine, Klinikum rechts der Isar, Technical University of Munich, Munich, Germany.
3Department of Internal Medicine, Hallym University College of Medicine, Chuncheon, Republic of Korea.
4Institute for Liver and Digestive Diseases, Hallym University, Chuncheon, Republic of Korea.
Corresponding author:
Ariel E. Feldstein, MD, Professor of Pediatrics, University of California San Diego
3020 Children’s Way, MC 5030, San Diego, CA, 92103-8450
Tel: (858) 966 – 8907
Email: afeldstein@ucsd.edu

## Slide 2
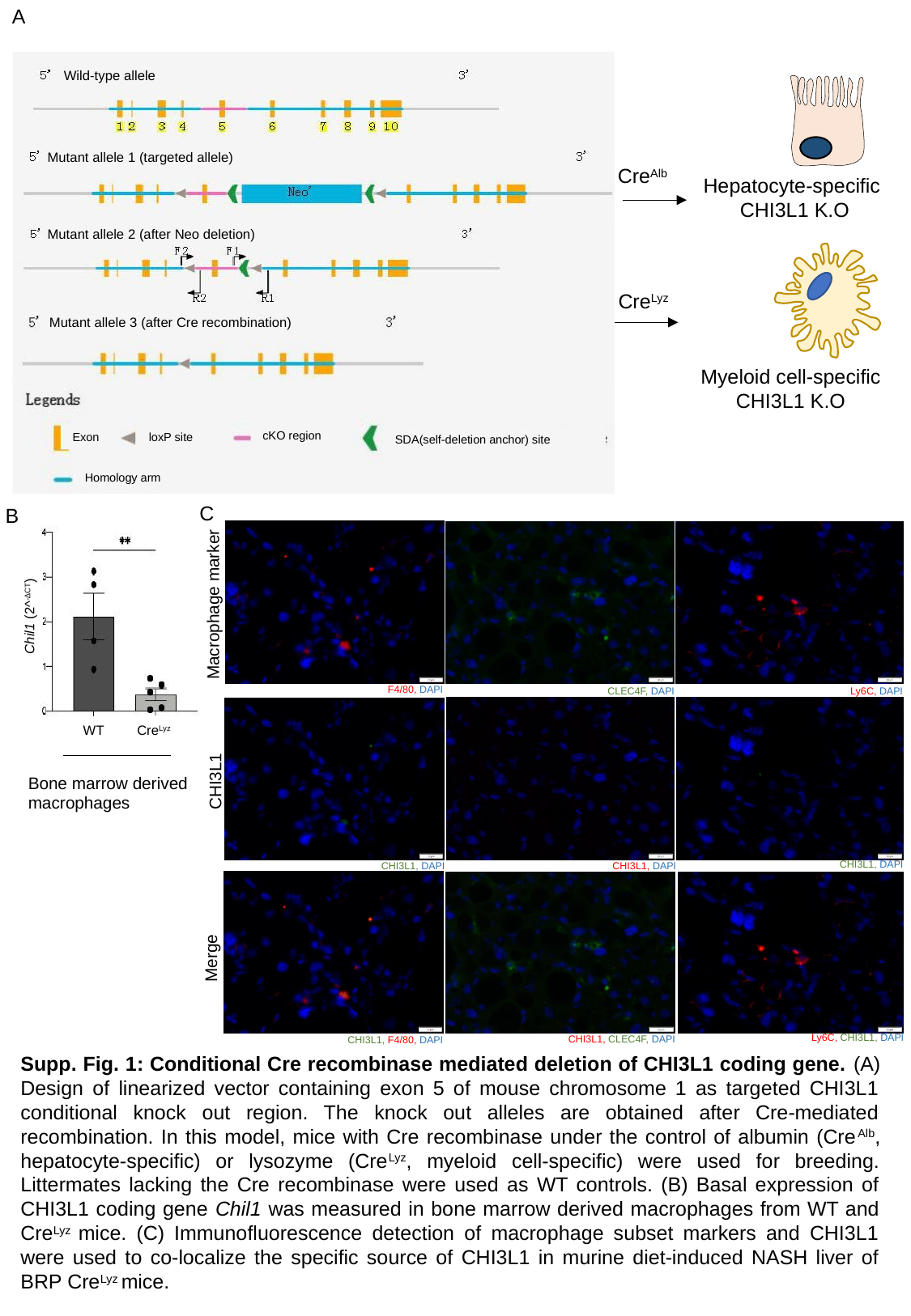

A
Wild-type allele
Mutant allele 1 (targeted allele)
Mutant allele 2 (after Neo deletion)
Mutant allele 3 (after Cre recombination)
CreAlb
Hepatocyte-specific
CHI3L1 K.O
CreLyz
Myeloid cell-specific CHI3L1 K.O
cKO region
Exon
loxP site
SDA(self-deletion anchor) site
Homology arm
C
B
Chil1 (2^-∆CT)
CreLyz
WT
Bone marrow derived macrophages
Macrophage marker
F4/80, DAPI
Ly6C, DAPI
CLEC4F, DAPI
CHI3L1
CHI3L1, DAPI
CHI3L1, DAPI
CHI3L1, DAPI
Merge
Ly6C, CHI3L1, DAPI
CHI3L1, CLEC4F, DAPI
CHI3L1, F4/80, DAPI
Supp. Fig. 1: Conditional Cre recombinase mediated deletion of CHI3L1 coding gene. (A) Design of linearized vector containing exon 5 of mouse chromosome 1 as targeted CHI3L1 conditional knock out region. The knock out alleles are obtained after Cre-mediated recombination. In this model, mice with Cre recombinase under the control of albumin (CreAlb, hepatocyte-specific) or lysozyme (CreLyz, myeloid cell-specific) were used for breeding. Littermates lacking the Cre recombinase were used as WT controls. (B) Basal expression of CHI3L1 coding gene Chil1 was measured in bone marrow derived macrophages from WT and CreLyz mice. (C) Immunofluorescence detection of macrophage subset markers and CHI3L1 were used to co-localize the specific source of CHI3L1 in murine diet-induced NASH liver of BRP CreLyz mice.

## Slide 3
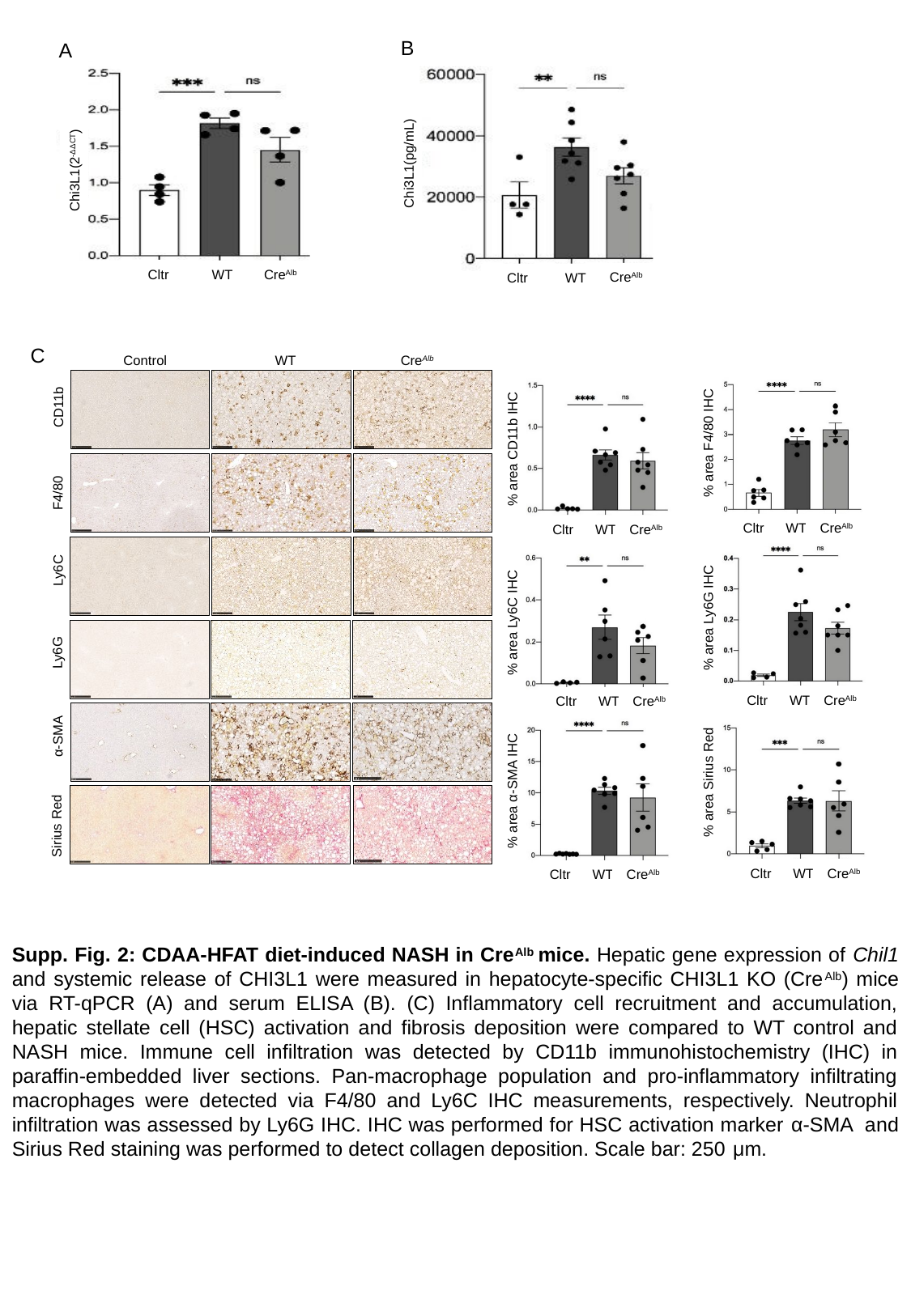

B
A
Cltr
WT
CreAlb
CreAlb
Cltr
WT
Chi3L1(pg/mL)
CHI3L1 (pg/mL)
Chi3L1(2-ΔΔCT)
Chil1 (2^-∆CT)
C
Control
CreAlb
WT
CD11b
% area F4/80 IHC
% area CD11b IHC
F4/80
Cltr
WT
CreAlb
Cltr
WT
CreAlb
Ly6C
% area Ly6G IHC
% area Ly6C IHC
Ly6G
Cltr
WT
CreAlb
Cltr
WT
CreAlb
α-SMA
% area Sirius Red
% area α-SMA IHC
Sirius Red
Cltr
WT
CreAlb
Cltr
WT
CreAlb
Supp. Fig. 2: CDAA-HFAT diet-induced NASH in CreAlb mice. Hepatic gene expression of Chil1 and systemic release of CHI3L1 were measured in hepatocyte-specific CHI3L1 KO (CreAlb) mice via RT-qPCR (A) and serum ELISA (B). (C) Inflammatory cell recruitment and accumulation, hepatic stellate cell (HSC) activation and fibrosis deposition were compared to WT control and NASH mice. Immune cell infiltration was detected by CD11b immunohistochemistry (IHC) in paraffin-embedded liver sections. Pan-macrophage population and pro-inflammatory infiltrating macrophages were detected via F4/80 and Ly6C IHC measurements, respectively. Neutrophil infiltration was assessed by Ly6G IHC. IHC was performed for HSC activation marker α-SMA and Sirius Red staining was performed to detect collagen deposition. Scale bar: 250 μm.

## Slide 4
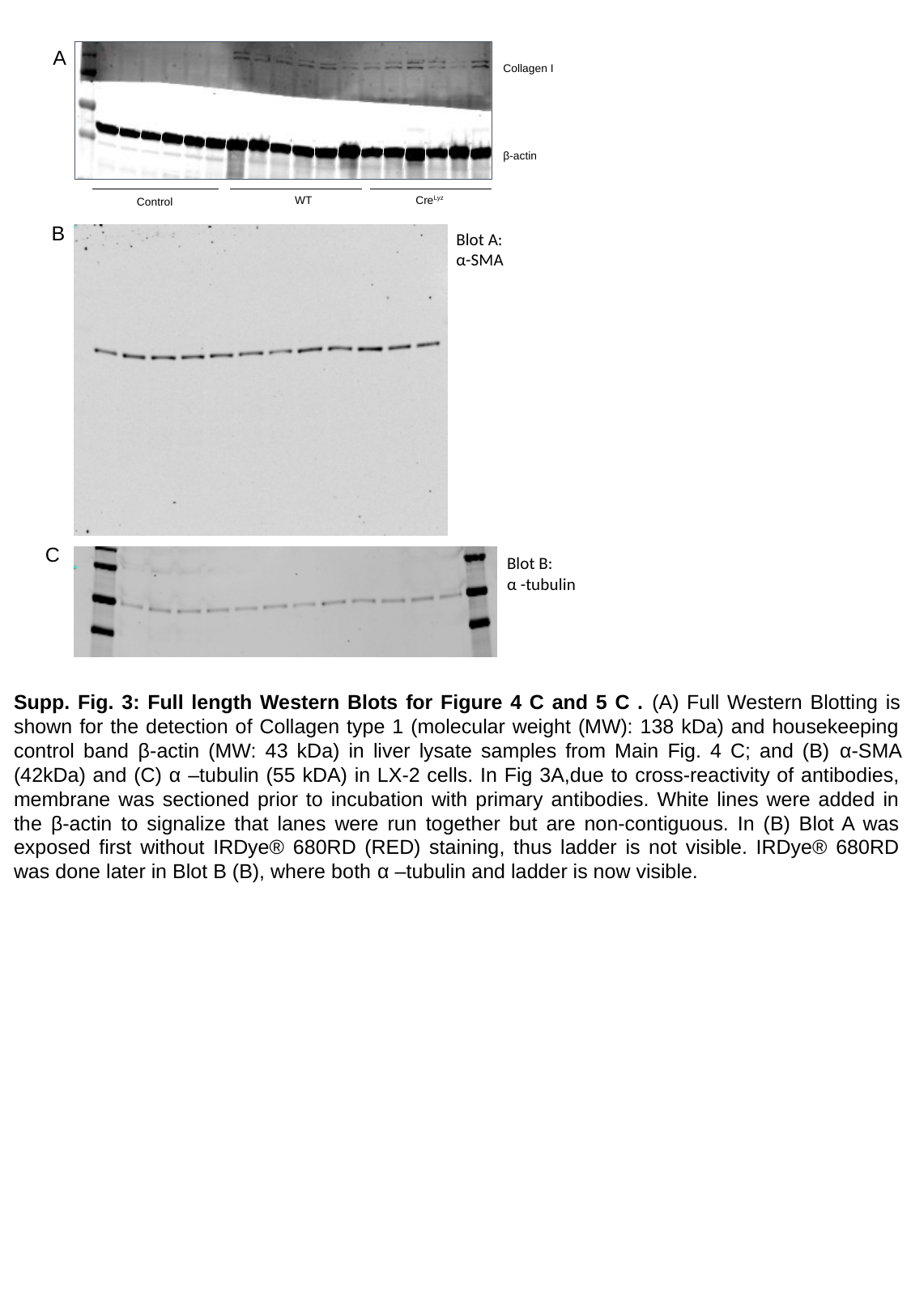

A
Collagen I
β-actin
WT
CreLyz
Control
B
Blot A:
α-SMA
C
Blot B:
α -tubulin
Supp. Fig. 3: Full length Western Blots for Figure 4 C and 5 C . (A) Full Western Blotting is shown for the detection of Collagen type 1 (molecular weight (MW): 138 kDa) and housekeeping control band β-actin (MW: 43 kDa) in liver lysate samples from Main Fig. 4 C; and (B) α-SMA (42kDa) and (C) α –tubulin (55 kDA) in LX-2 cells. In Fig 3A,due to cross-reactivity of antibodies, membrane was sectioned prior to incubation with primary antibodies. White lines were added in the β-actin to signalize that lanes were run together but are non-contiguous. In (B) Blot A was exposed first without IRDye® 680RD (RED) staining, thus ladder is not visible. IRDye® 680RD was done later in Blot B (B), where both α –tubulin and ladder is now visible.

## Slide 5
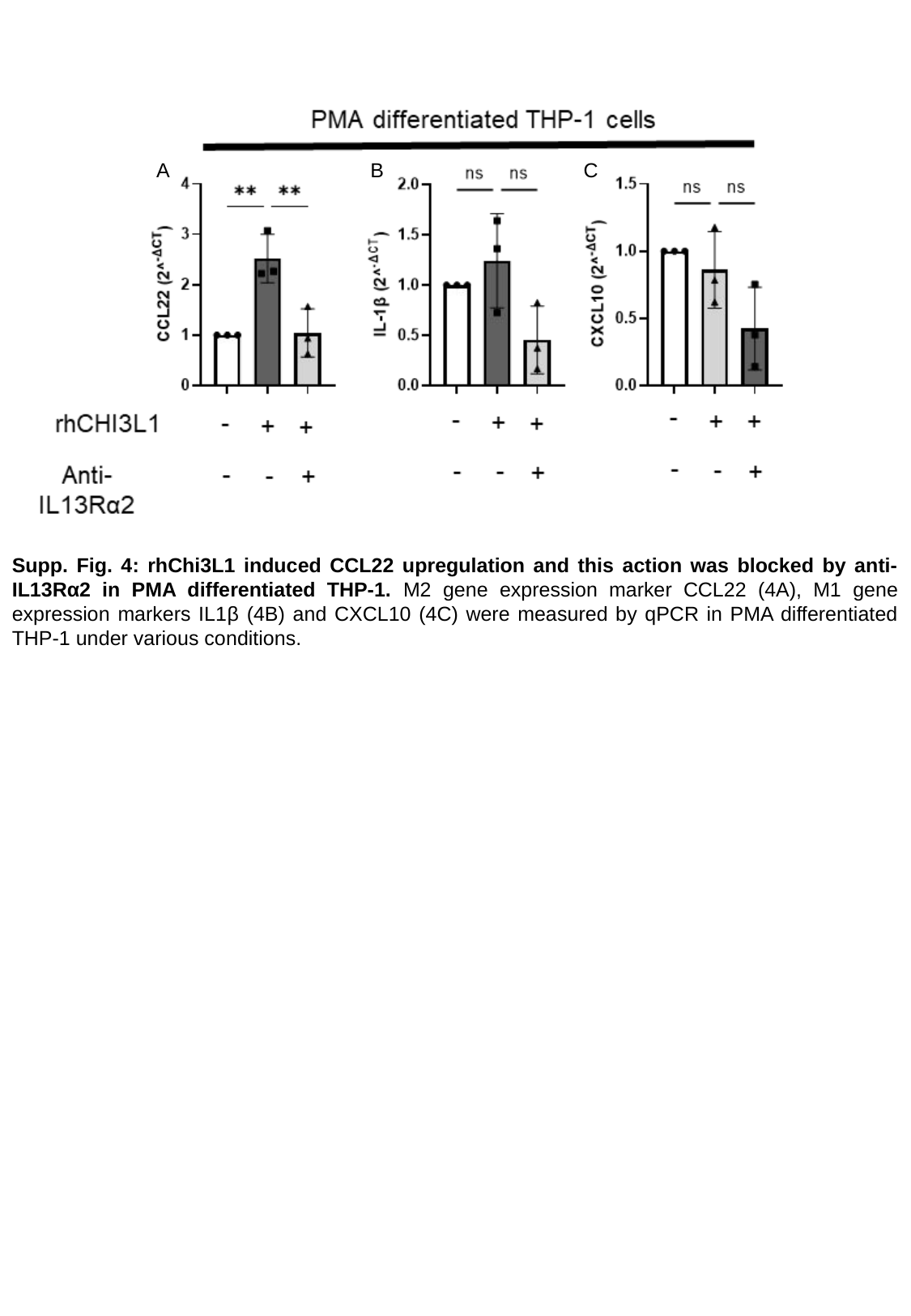

A
B
C
Supp. Fig. 4: rhChi3L1 induced CCL22 upregulation and this action was blocked by anti-IL13Rα2 in PMA differentiated THP-1. M2 gene expression marker CCL22 (4A), M1 gene expression markers IL1β (4B) and CXCL10 (4C) were measured by qPCR in PMA differentiated THP-1 under various conditions.
